# Evaluation of the Burden of Unsuspected Pulmonary Tuberculosis and Co-Morbidity with Non-Communicable Diseases in Sputum Producing Adult Inpatients

**DOI:** 10.1371/journal.pone.0040774

**Published:** 2012-07-27

**Authors:** Matthew Bates, Justin O’Grady, Peter Mwaba, Lophina Chilukutu, Judith Mzyece, Busiku Cheelo, Moses Chilufya, Lukundo Mukonda, Maxwell Mumba, John Tembo, Mumba Chomba, Nathan Kapata, Andrea Rachow, Petra Clowes, Markus Maeurer, Michael Hoelscher, Alimuddin Zumla

**Affiliations:** 1 Division of Infection and Immunity, Department of Infection, University College London, London, United Kingdom; 2 University of Zambia and University College London Medical School (UNZA-UCLMS) Research and Training Project, University Teaching Hospital, Lusaka, Zambia; 3 Ministry of Health, Lusaka, Zambia; 4 National Tuberculosis Control Programme, Ministry of Health, Lusaka, Zambia; 5 Mbeya Medical Research Programme (MMRP), Mbeya, Tanzania; 6 Department of Microbiology, Tumour and Cell Biology, Karolinska Institute, Stockholm, Sweden; 7 Department for Infectious Diseases and Tropical Medicine, Klinikum of the University of Munich, Munich, Germany; Institut de Pharmacologie et de Biologie Structurale, France

## Abstract

**Background:**

A high burden of tuberculosis (TB) occurs in sub-Saharan African countries and many cases of active TB and drug-resistant TB remain undiagnosed. Tertiary care hospitals provide an opportunity to study TB co-morbidity with non-communicable and other communicable diseases (NCDs/CDs). We evaluated the burden of undiagnosed pulmonary TB and multi-drug resistant TB in adult inpatients, regardless of their primary admission diagnosis, in a tertiary referral centre.

**Methodology/Principal Findings:**

In this prospective study, newly admitted adult inpatients able to produce sputum at the University Teaching Hospital, Lusaka, Zambia, were screened for pulmonary TB using fluorescent smear microscopy and automated liquid culture. The burden of pulmonary TB, unsuspected TB, TB co-morbidity with NCDs and CDs was determined. Sputum was analysed from 900 inpatients (70.6% HIV infected) 277 (30.8%) non-TB suspects, 286 (31.8%) TB suspects and 337 (37.4%) were already receiving TB treatment. 202/900 (22.4%) of patients had culture confirmed TB. TB co-morbidity was detected in 20/275 (7.3%) NCD patients, significantly associated with diabetes (P = 0.006, OR 6.571, 95%CI: 1.706–25.3). 27/202 (13.4%) TB cases were unsuspected. There were 18 confirmed cases of MDR-TB, 5 of which were unsuspected.

**Conclusions/Significance:**

A large burden of unsuspected pulmonary TB co-morbidity exists in inpatients with NCDs and other CDs. Pro-active sputum screening of all inpatients in tertiary referral centres in high TB endemic countries is recommended. The scale of the problem of undiagnosed MDR-TB in inpatients requires further study.

## Introduction

The WHO estimates that In 2010, there were 1.45 million TB-related deaths, with the highest burden of tuberculosis (TB) in sub-Saharan Africa (SSA), where many cases of active TB and drug-resistant TB remain undiagnosed [1]. These TB-related deaths often occur at tertiary referral centres, which concentrate a broad range of critically ill patients where the primary admission diagnosis is the focus of medical attention. TB screening programmes in SSA have traditionally been more community based and focussed on primary and secondary care facilities [2]. TB cases that are missed at tertiary referral centres in SSA, may be to some degree symptomatic, but overlooked with the focus of attention on the main admission symptoms and referral diagnoses. Missed cases might also result from subclinical/asymptomatic/incipient TB, although the definitions for these terms are not yet clear [3], [4].

Subclinical TB has been defined as ‘asymptomatic disease in immunocompromised hosts largely associated with loss of containment’ [3] and this term has commonly been used in studies of asymptomatic TB HIV positive cohorts [4], [5], [6], [7], [8], [9], [10] where active disease is detected through sputum culture [11]. ‘Incipient TB’ has been defined as ‘contained disease in asymptomatic, relatively immunocompetent persons’ [3] although broader cohorts which include HIV negative patients, and those with NCDs, have been less well studied. SSA is facing a growing NCD burden with diseases such as cardiovascular disease, diabetes mellitus, chronic respiratory disease and cancers contributing up to 25% of deaths [12], [13], [14], [15]. There is a growing awareness of the influence of TB co-morbidity with some NCDs (such as smoking related lung disease, renal disorders, diabetes, malnutrition, alcohol and drug abuse) [16], [17] and understanding this is increasingly becoming important for TB control [1], [18]. MDR-TB continues to pose a major threat globally, and due to poor surveillance and testing facilities in developing countries [19], less than 2% of new TB cases and 6% of retreatment cases being tested for resistance [1]. Referral centres may concentrate cases of MDR-TB and if these drug resistant patients are not promptly diagnosed, isolated and appropriately treated, they pose a major transmission risk to other patients, hospital staff and visitors.

Zambia has a high TB incidence (462/100,000 population) [1] but no routine data are collected for MDR-TB and no confirmed cases were reported to the WHO in 2010 [1], [20]. National guidelines recommend pulmonary TB screening (using sputum smear microscopy, and automated liquid culture at referral centres) only in TB suspects: patients who present with ‘a persistent cough for more than 2 weeks’ [21]. These guidelines are broadly applied at both primary and tertiary centres. Patients with subclinical/asymptomatic/incipient TB or those with intermittent symptoms, and those with a more acute co-morbidity, are possibly missed by this programme. The University Teaching Hospital (UTH) in Lusaka is Zambia’s main referral centre, with its internal medicine department receiving an estimated 6000 adult admissions per year (mortality at 183/1000 admissions). Tertiary care hospitals like UTH provide an opportunity to study asymptomatic TB, and TB co-morbidity with NCDs and other CDs. We evaluated the burden of pulmonary TB and multi-drug resistant TB in adult inpatients, regardless of their primary admission diagnosis, in a tertiary referral centre in Zambia.

## Methods and Study Population

### Ethics Approval

This study was approved by the research ethics review committee of the University of Zambia School of Medicine, Lusaka, Zambia. All study participants gave written informed consent and the study was conducted in accordance with ethics committee guidelines.

### Study Design and Setting

A prospective study to assess the burden of pulmonary TB, MDR-TB, unsuspected TB and co-morbidity with NCDs and CDs other than TB, irrespective of admission diagnosis and HIV status, in adult inpatients presenting to UTH, Lusaka, Zambia - a tertiary referral centre.

### Definitions

#### PTB

Pulmonary tuberculosis.

#### MDR-TB

TB caused by *Mycobacterium tuberculosis* (*M.tb*) strains resistant to at least isoniazid and rifampicin.

#### TB suspect

Patient with presence of cough on admission, of at least 2 weeks’ duration (Zambia National guidelines) [21].

#### Non-TB suspect

Any patient who is not a TB suspect in accordance with the above definition.

#### Current TB Patient

Patient currently on TB therapy, initiated prior to admission.

#### Unsuspected TB

Culture confirmed TB in a non-TB suspect. As our cohort contains a broad range of patients, including HIV negative, HIV positive (at different stages of immunosuppression) and NCD patients, we use the term ‘unsuspected TB’ to define all culture confirmed TB in patients in whom TB was not suspected. These may include subclinical, asymptomatic, incipient and intermittent cases, as well as symptomatic cases missed due to co-morbidities.

#### Communicable Diseases (CDs)

Infectious diseases that can be transmitted between people.

#### Non-communicable Disease (NCDs)

Diseases that cannot be transmitted between people.

#### TB co-morbidity

Presence of culture confirmed TB, presenting with a different disease.

### Patient Population and Recruitment

New adult inpatient admissions to UTH were approached irrespective of admission diagnosis, including TB suspects, non-TB suspects and current TB patients, and informed consent was obtained from those willing to participate. The sole inclusion criterion was that they could produce at least one sputum specimen for analysis. Clinical details including the admission diagnosis which necessitated hospital admission were recorded. Sputum samples were collected. Due to the very high HIV prevalence in the population, the hospital has in place a Diagnostic Counselling and Testing (DCT) scheme. The majority of patients enrolled in the study were tested for HIV, as part of routine practise on admission. To assess to what degree our cohort of sputum producers was representative of the general hospital population, we compared our cohort with a set of 960 hospital records accounting for all adult admissions over a 2 month period.

### Sample Collection

In accordance with routine hospital protocols, clinical staff supervised the collection of spot sputum from patients, and then left containers to collect up to two other specimens over the next 24 hours. Sputum induction is not routine at the hospital and was not performed.

### Microscopy, Sputum Culture and Phenotypic Drug Susceptibility Testing (DST)

Fluorescent smear microscopy was performed directly on sputum specimens as described previously [22]. For culture, sputum specimens (2–10 ml) were homogenised and digested in NALC-NaOH (1.5% final concentration), and vortexed for 30 seconds at 5 minutes intervals for 15 minutes. Samples were then concentrated at 4000×g for 15 minutes, the supernatant was removed and sediment re-suspended in 5 mls phosphate buffer (pH 6.8), irrespective of the original sample volume. The resulting suspension was used to perform Mycobacterial Growth Indicator Tube (MGIT - BD, Franklin Lakes, NJ, USA) culture. One MGIT tube was inoculated with 0.5 ml concentrated sputum and incubated in the BACTEC 960 system (BD, Franklin Lakes, NJ, USA). For patients who submitted 2 or 3 sputum specimens, the most mucoid was used for culture. Cultures were considered negative if no growth was observed after 42 days. Positive MGIT cultures were confirmed as containing *M.tb* complex with no growth on blood agar plates and a positive TBcID (BD) culture confirmation test. Contaminated samples were retreated and re-cultured and excluded if still contaminated. Phenotypic DST was performed on *M.tb* positive cultures using the BACTEC MGIT 960 SIRE kit (BD, Franklin Lakes, NJ, USA) according to the manufacturer’s instructions.

### Data Management and Analysis

Clinical and laboratory data were compiled in databases using double data entry and Epidata software [23]. Selected variables were exported to SPSS v 18 (IBM, Armonk, NY, USA) for analysis. Univariate comparisons of the proportions of different patient groups with the hospital population were compared by chi-squared test. Univariate and multivariate analysis for factors effecting TB burden in different patient groups was performed using binary logistic regression.

## Results

### Study Cohort

**Figure 1:**
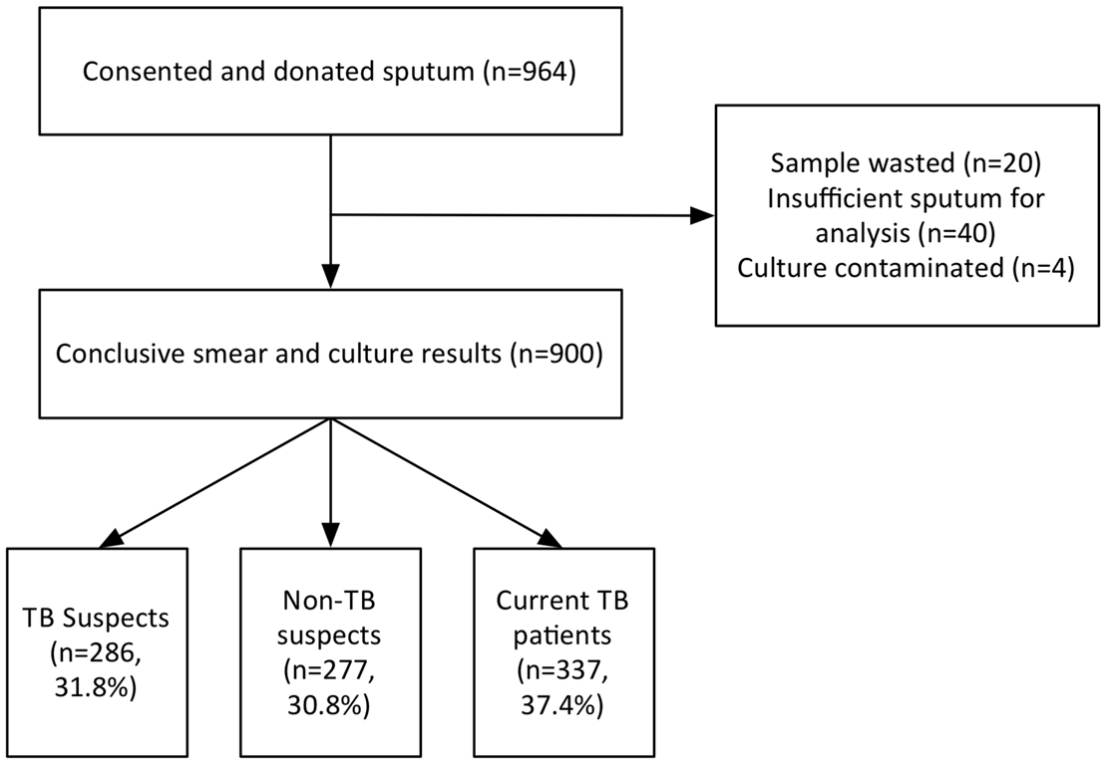
Patient Recruitment Summary.

During the 15 month period from Sept 2010 to Dec 2011, we conducted 218 days of recruitment with a median of 4 recruits per day. A total of 964 patients were enrolled, from whom smear and culture results were obtained for 900 patients and analysed (Figure 1). A total of 31.8% (286/900) were TB suspects based on the Zambian National TB Guidelines [21]), 37.4% (337/900) were current TB patients on treatment, and 30.8% (277/900) were non-TB suspects. Comparison with the overall hospital population (data for all admissions collected from hospital records over a 2 month period) showed that the proportion of current TB patients and TB suspects in our study cohort was significantly greater (1.7 and 3.6 fold respectively  =  P<0.0001) than the general inpatient population and that patients not suspected of having TB were significantly under-represented (0.4 fold, P<0.0001) (Table 1) as expected (due the recruitment requirement of sputum production). This illustrates that sputum can be successfully collected and analysed for TB, from inpatients admitted with a variety of conditions, not only those suspected of TB (or other pulmonary conditions).

**Table 1:** Study population demographics.

	Hospital Population (n = 960)	Study(n = 900)	Significance^a^
**Median (IQR) age (Years)**	36 (29–48)	35 (28–43)	
**Sex (Male)**	49.4% (471/954)	50.2% (452/898)	P = 0.679
**HIV infection**	63.1% (536/849)	67.3% (606/858)	P = 0.001
**TB status**			
** Current TB**	22.4% (214/957)	37.4% (337/900)	P<0.001
** TB suspects**	8.8% (84/957)	31.8% (286/900)	P<0.001
** Non-TB suspects**	68.7% (659/957)	30.8% (277/900)	P<0.001
**Admission Diagnosis**	n = 946^b^		
** Respiratory Disorders (excluding TB)**	95 (10.0%)	155 (17.2%)	P<0.001
** PTB**	72 (7.6%)	176 (19.6%)	P<0.001
** EPTB**	54 (5.7%)	56 (6.2%)	P = 0.641
** CNS disorders**	156 (16.5%)	92 (10.2%)	P = 0.001
** Cancer**	33 (3.5%)	40 (4.4%)	P = 0.292
** Cardiac Disorders**	166 (17.5%)	125 (13.9%)	P = 0.031
** Gastrointestinal Disorders**	70 (7.4%)	58 (6.4%)	P = 0.419
** Metabolic Disorders**	100 (10.6%)	37 (4.1%)	P<0.001
** Renal Disorders**	38 (4.0%)	47 (5.2%)	P = 0.217
** Diabetes**	57 (6.0%)	19 (2.1%)	P<0.001
** Other**	105 (11.1%)	94 (10.4%)	P = 0.650

IQR – interquartile range; TB – tuberculosis; PTB – pulmonary TB; EPTB - extrapulmonary TB; CNS – central nervous system.

a Pearson chi-squared test.

b Admission diagnosis could not be gathered from 14 admissions.

### Unsuspected TB and TB Co-morbidity with HIV

**Figure 2:**
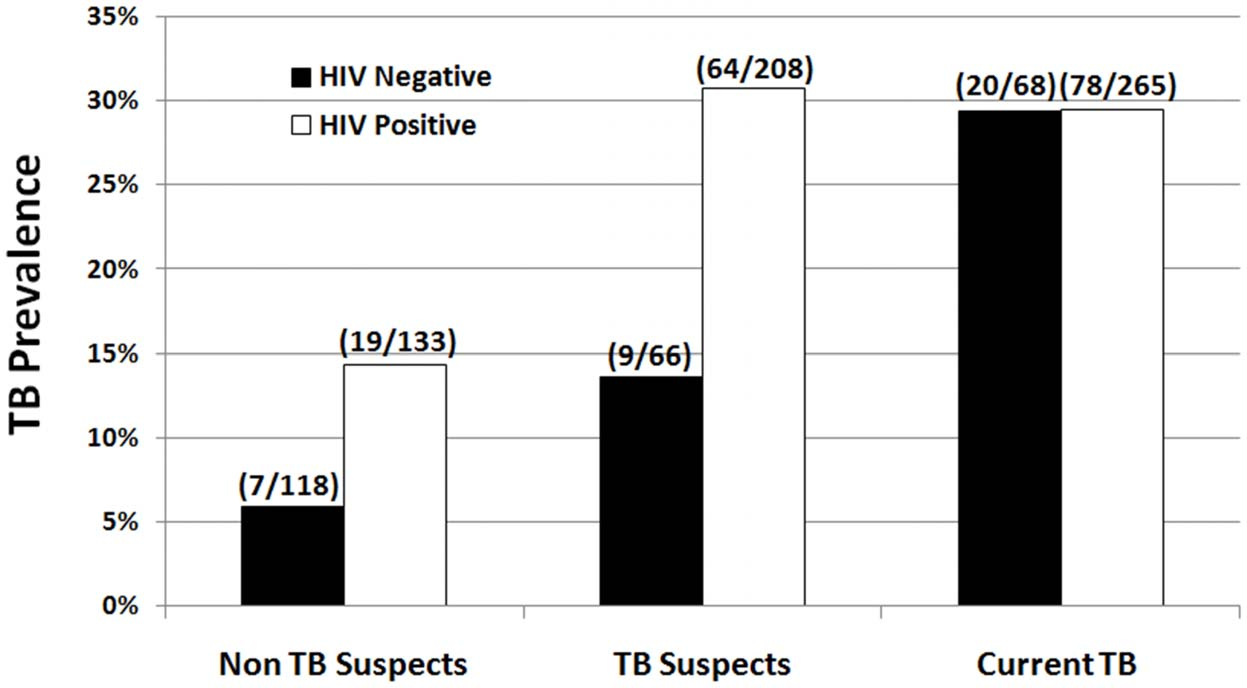
TB prevalence in different patient groups stratified by HIV status (HIV status was available for 858/900 study participants).

22.4% (202/900) of patients recruited had culture confirmed TB (Table 2). Univariate regression analysis showed that TB burden did not differ by gender, but was significantly more likely in HIV infected patients (P<0.001, OR 2.171 (95%CI: 1.461–3.022)). HIV prevalence within the cohort was 70.6%, and 82% (161/197) of all culture confirmed TB cases detected were in HIV positive patients. With respect to age, there was an annual 2.6% decrease in the likelihood of TB (P<0.001, OR 0.974 (95% CI: 0.961-0.988)). The burden of culture confirmed TB in both current TB patients and TB suspects was similar (29.4% (99/337) and 26.6% (76/286) respectively). Interestingly, 9.7% (27/277) of non-TB suspects also had culture confirmed active TB (Figure 2), accounting for 13.4% of all culture confirmed TB cases. Of these patients, five had a history of TB treatment. Furthermore, five of eight renal and two of four diabetic culture confirmed TB patients were from this unsuspected group.

### Admission Diagnosis and TB Co-morbidity with other Communicable and Non-communicable Diseases

275 patients showed clear evidence of admission diagnosis of co-morbidity with a non-communicable disease. Likewise, 306 patients presented with communicable diseases (excluding TB and HIV) (Table 2). Admission diagnoses of PTB or EPTB and those that could not be categorically assigned to either group (eg. anaemia) were excluded from this analysis. 20/275 (7.3%) patients with NCDs, and 74/306 (24.2%) patients with CDs were found to have active TB. Binary logistic regression analysis, controlling for the effects of HIV and age, shows that among the NCD patient group, the burden of TB was significantly greater in diabetes patients (P  = 0.025, OR 6.571 [95%CI: 1.706–25.302]) patients, although due to the small numbers in these patient categories, the confidence intervals are broad. Within the CD patients, TB co-morbidities were as prevalent among gastrointestinal and CNS patients, as among respiratory patients, showing that TB co-morbidity should be considered in patients with respiratory and non-respiratory infections (Table 2).

### Culture Confirmed MDR-TB

Culture DST results were available for 111 cases, of which 18 (16.2%) were MDR-TB. 33 sub-cultures were contaminated and 58 were not performed as DST was not available at the beginning of the study. All 18 MDR-TB cases detected were in current or suspected TB patients. Of the 18 MDR-TB cases, 12 were on or about to start relapse treatment. The remaining 6 MDR-TB cases were on their first course of treatment and 5 of these were not suspected by the attending physician.

**Table 2:** Burden of pulmonary TB and admission diagnosis co-morbidities with HIV, NCDs and CDs.

	Culture positive TB within sputum producers (n = 900)		Univariate Analysis		Multivariate Analysis^a^	
	Proportion (%)	95% CI	OR [95% CI]	Significance	OR [95% CI]	Significance
**Overall TB burden: 202/900 (22.4%)**						
**Gender**						
Female	106/446 (23.8%)	[20.0–28.1%]	-	-	-	-
Male	96/452 (21.2%)	[17.6–25.4%]	0.865 [0.632–1.184]	0.365	0.951 [0.687–1.316]	0.761
**HIV**						
HIV Negative	36/252 (14.3%)	[10.3–19.4%]	-	-	-	-
HIV Positive	161/606 (26.6%)	[23.1–30.3%]	2.171 [1.461–3.226]	<0.001	2.024 [1.356–3.022]	0.001
**Age Group** b			0.974 [0.961–0.988]	<0.001	0.979 [0.965–0.993]	0.004
15–30 yrs	84/312 (26.9%)	[22.2–32.2%]				
31–50 yrs	105/459 (22.9%)	[19.2–27.1%]				
51–70 yrs	12/98 (12.2%)	[6.8–20.8%]				
71–100 yrs	1/26 (3.7%)	[0.2–21.6%]				
**NCD co-morbidity with culture-positive tuberculosis: 20/275** c **(7.3% ) [4.6–11.2%]**						
Respiratory disorders	2/19 (10.5%)	[1.9–34.5%]	1.556 [0.333–7.267]	0.574	1.892 [0.392–9.138]	0.428
Renal Disorders	6/39 (15.4%)	[6.4–31.2%]	2.883 [1.036–8.027]	0.043	2.366 [0.827–6.766]	0.108
Diabetes	4/19 (21.1%)	[7.0–46.1%]	4.000 [1.189–13.46]	0.025	6.571 [1.706–25.30]	0.006
Cardiac Disorders	4/125 (3.2%)	[1.0–8.5%]	0.277 [0.090–0.851]	0.025	0.310 [0.096–1.001]	0.050
Cancer	1/40 (2.5%)	[0.1–14.7%]	0.291 [0.038–2.241]	0.236	0.193 [0.024–1.535]	0.120
**CD co-morbidity with culture-positive tuberculosis 74/306** d **(24.2%) [19.6–29.5%]**						
Respiratory infections[excluding TB ]	35/138 (25.4%)	[18.5–33.6%]	1.124 [0.665–1.899]	0.662	1.108 [0.648–1.894]	0.709
CNS Infections	15/67 (22.4%)	[13.5–34.5%]	0.880 [0.462–1.678]	0.698	0.894 [0.465–1.720]	0.737
Gastrointestinal Infections	13/46 (28.3%)	[16.5–43.7%]	1.285 [0.636–2.596]	0.484	1.253 [0.601–2.611]	0.547

Data are n TB positive/n tested (%) [95% CI], Odds Ratios (ORs) and associated confidence intervals (CIs) from binary logistic regression analysis.

a Multivariate analysis was controlled for the effects of Age and HIV.

b Age was analysed as a continuous variable but is displayed as grouped to illustrate the distribution.

c Three TB culture negative patients were represented in multiple NCD diagnosis categories.

d Two TB culture negative patients were represented in multiple CD diagnosis categories.

## Discussion

This study found a large burden of pulmonary TB in sputum producing inpatients at a high HIV burden tertiary referral centre in Zambia. The high TB case load (22.4%: 202/900) among patients able to expectorate was anticipated in a patient cohort with an HIV prevalence of 70.6%, where over 80% of all culture confirmed TB cases were associated with HIV. Despite moderate reductions in both HIV and TB prevalence in Zambia [2], [20], tertiary referral centres like UTH will continue to admit large numbers of HIV and TB/HIV co-infected patients for the foreseeable future. In this study there were three other key findings: a) 27 out of 202 (13.4%) TB cases were unsuspected on admission and would have remained undiagnosed if not actively screened on this study; b) A total of 94 TB cases presented as co-morbidity with other diseases, 20 of which were NCDs; c) 18 out of 111 (16.2%) TB cases tested were MDR-TB and 5 out of these 18 (27.8%) MDR-TB cases were not suspected in the differential diagnosis on admission. These findings highlight that many TB and MDR-TB cases remain undiagnosed, and that passive case finding using current clinical criteria outlined in the Zambia National TB Program national guidelines for investigating suspected TB cases, are inadequate for use in inpatients at UTH. Furthermore, these findings raise concerns over TB and MDR-TB transmission within the hospital and highlight the need for more focussed investments into active screening and surveillance for both TB and MDR-TB.

This study was uniquely designed to focus not only on screening of inpatients who were suspected of having active pulmonary TB on admission by the admitting physician, but also to determine the extent to which TB cases were being missed, and the burden of TB co-morbidity with NCDs and CDs. We recruited roughly equal numbers of TB suspects, non-TB suspects and current TB patients on treatment. The relative proportions of these three groups to be recruited were not pre-determined, with study clinical officers instructed to approach all admissions. That roughly one third of the cohort were not suspected of TB, yet able to expectorate and recruited onto the study was surprising, and shows that a broader range of patients than you might expect, can expectorate and could be readily screened for TB. Across the cohort as a whole, a broad range of patients were recruited, with 75.3% (678/900) of patients having an admission diagnosis other than TB, including NCDs and CDs.

Several African studies have reported HIV-associated subclinical TB infections in which patients do not present with any symptoms of active TB [4], [8], [11]. In this study, the cohort contained both HIV positive and negative patients, and those with a broad range of co-morbidities with other diseases, and so we use the term ‘unsuspected TB’ to define all culture confirmed TB cases in patients in whom TB was not suspected at admission. These unsuspected cases accounted for 13.4% (27/202) of all culture confirmed TB cases. Some of these cases may have been identified if more rigorous symptom based case definitions were available, with improved clinical awareness of the possibility of TB in all inpatients, coupled with better trained and less over-worked staff, and improved access to good quality radiography and laboratory services. Likewise, some of these unsuspected cases may fall into proposed definitions for subclinical or incipient TB [3]. Whether subclinical, incipient or broadly symptomatic but overlooked due to co-morbidity with other diseases, a simple screening policy for all inpatients who can expectorate, using the locally available screening services, would detect these cases. 37% (10/27) of unsuspected cases were smear positive by microscopy, so even in centres where culture services are not available, significant numbers of cases could be captured with this policy.

We showed, as seen in other SSA countries, that HIV positive patients are twice as likely to have active pulmonary TB, with two thirds of TB patients co-infected with HIV [24]. Many TB/HIV patients also have NCDs or opportunistic CDs. With respect to unsuspected TB, HIV was no more prevalent in the unsuspected, than in suspected TB cases, showing that in both HIV positive and negative patients, classical symptoms of pulmonary TB may be absent or overlooked [25].

The growing problem of NCDs in SSA is well documented [12], [13], [14], [15], but there are only few reports that have addressed TB co-morbidity with NCDs and CDS other than TB [1], [18], [24]. In this study, roughly half the TB cases were co-morbidities with other NCDs and CDs. Within the NCD group, there was a significantly higher likelihood of TB co-morbidity in patients with renal disorders and those with diabetes compared to other NCD patients, an association that has been documented elsewhere [17], [18]. These two patient groups also featured prominently in the unsuspected TB cases, indicating that these NCD presentations are possibly masking TB. Amongst 306 patients with a CD admission diagnoses other than TB, culture confirmed TB was detected in 74 patients (24.2%), and interestingly, TB was no more commonly detected in patients with other respiratory infections, verses those with CNS or gastrointestinal infections and the likelihood of culture confirmed TB was no greater in respiratory patients in both univariate and multivariate analysis (controlling for the effects of HIV and age). This demonstrates that TB can underlie a broad range of infectious diseases and screening programmes should not just focus on respiratory patients.

Our data show that MDR-TB and unsuspected MDR-TB are significant problems in inpatients at UTH. In our study, of those culture positive cases analysed by culture DST, 18 out of 111 (16.2%) had MDR-TB and 5 were undiagnosed, on inappropriate therapy with first line TB treatment, and were not suspected of MDR-TB by the attending physician. This represents a significant failure to adequately diagnose and treat MDR-TB, putting other patients and staff at unnecessary risk. A more pro-active routine screening program for TB and MDR-TB is required. This finding is confluent with the fact that MDR-TB surveillance in Zambia is poor, with MDR-TB data not being routinely collected in hospitals or primary health care facilities, due to under-funding and inadequate laboratory services [1], [2], [20].

As this was a prospective descriptive study to evaluate the burden of TB in sputum producing new inpatient admissions using diagnostics that require disease-associated specimens, our patient cohort is not representative of the broader hospital population. Through comparison with hospital admissions data, we confirmed that patients with respiratory illnesses and TB patients were over represented and so we make no claim about the prevalence of TB within the general inpatient population. There were also factors which could have contributed to missing some TB cases. Performing culture on multiple specimens, using sputum induction and extensive investigations for extrapulmonary TB would have likely yielded more cases. Despite these limitations, the high TB burden (diagnosed and undiagnosed); co-morbidity with NCDs; and the identification of MDR-TB in inpatients able to produce sputum, calls for more pro-active screening for TB in medical inpatients using routine diagnostic protocols already in place.

Most countries in SSA are now moving away from vertical programmes for TB and HIV services and more emphasis is on merging TB/HIV services for delivery of joint care [2], [26]. The significant number of unsuspected TB cases seen here, missed in part due to co-morbidity with NCDs, calls for more pro-active screening of all inpatient admissions locally, and should be considered by other hospitals in the region. The majority of unsuspected cases detected did not have a history of TB. These un-detected active TB cases are being admitted to a tertiary referral centre, where 70% of inpatients are HIV positive, presenting a high transmission risk. With increasing awareness of the co-morbidity between NCDs and TB, it would be appropriate to incorporate a unified health service to deal with all diseases affecting the population. Within this programme, pro-active screening for TB in those seeking care, capable of producing sputum, is appropriate in light of our findings.
